# AGI grade-guided chaiqin chengqi decoction treatment for predicted moderately severe and severe acute pancreatitis (CAP trial): study protocol of a randomised, double-blind, placebo-controlled, parallel-group, pragmatic clinical trial

**DOI:** 10.1186/s13063-022-06792-x

**Published:** 2022-11-08

**Authors:** Zhiyao Chen, Xiaonan Yang, Jia Guo, Tao Jin, Ziqi Lin, Ping Zhu, Jing Li, Ling Li, Xin Sun, Dan Du, Kun Jiang, Yanqiu He, Fei Cai, Lan Li, Cheng Hu, Qingyuan Tan, Wei Huang, Lihui Deng, Qing Xia

**Affiliations:** 1 West China Centre of Excellence for Pancreatitis, Institute of Integrated Traditional Chinese and Western Medicine, West China Hospital, Sichuan University, Chengdu, China; 2 Chinese Evidence-based Medicine Centre, Cochrane China Centre and MAGIC China Centre, West China Hospital, Sichuan University, Chengdu, China; 3 West China-Washington Mitochondria and Metabolism Centre, West China Hospital, Sichuan University, Chengdu, China; 4 Institutes for Systems Genetics, Frontiers Science Centre for Disease-related Molecular Network, West China Hospital, Sichuan University, Chengdu, China; 5 Liverpool Pancreatitis Research Group, Institute of Systems, Molecular and Integrative Biology, University of Liverpool and Liverpool University Hospitals NHS Foundation Trust, University of Liverpool, L69 3GE, Liverpool, UK

**Keywords:** Acute pancreatitis, Chaiqin chengqi decoction, Randomised controlled trial, Traditional Chinese medicine

## Abstract

**Background:**

Acute pancreatitis (AP) is a common digestive disease with increased incidence globally but without internationally licenced pharmacological therapy. Moderately severe and severe acute pancreatitis (MSAP/SAP) contributes predominately for its morbidities and mortality and has been managed in West China Hospital for decades using the traditional Chinese medicinal formula chaiqin chengqi decoction (CQCQD). The current study tests whether the early administration of CQCQD will result in improved clinical outcomes in predicted MSAP/SAP patients.

**Methods:**

This is a single-centre, randomised, controlled, double-blind pragmatic clinical trial. AP patients aged 18–75 admitted within 72 h of onset will be assessed at admission for enrolment. We excluded the predicted mild acute pancreatitis (Harmless Acute Pancreatitis Score > 2 at admission) and severe organ failure (Sequential Organ Failure Assessment [SOFA] score of respiratory, cardiovascular, or renal systems > 3) at admission. Eligible patients will be randomly allocated on a 1:1 basis to CQCQD or placebo control administration based on conventional therapy. The administration of CQCQD and placebo is guided by the Acute Gastrointestinal Injury grade-based algorithm. The primary outcome measure will be the duration of respiratory failure (SOFA score of respiratory system ≥ 2) within 28 days after onset. Secondary outcome measures include occurrence of new-onset any organ failure (SOFA score of respiratory, cardiovascular, or renal system ≥ 2) and new-onset persistent organ failure (organ failure lasts > 48 h), dynamic surrogate biochemical markers and clinical severity scores, gut-centred treatment modalities, local complications status, intensive care need and duration, surgical interventions, mortality, and length of hospital stay. Follow-up will be scheduled on 6, 12, and 26 weeks after enrolment to assess AP recurrence, local complications, the requirement for surgical interventions, all-cause mortality, and patient-reported outcomes.

**Discussion:**

The results of this study will provide high-quality evidence to appraise the efficacy of CQCQD for the early management of AP patients.

**Trial registration:**

Chictr.org.cn Registry (ChiCTR2000034325). Registered on 2 July, 2020.

**Supplementary Information:**

The online version contains supplementary material available at 10.1186/s13063-022-06792-x.

## Administrative information

Note: the numbers in curly brackets in this protocol refer to SPIRIT checklist item numbers. The order of the items has been modified to group similar items (see http://www.equator-network.org/reporting-guidelines/spirit-2013-statement-defining-standard-protocol-items-for-clinical-trials/)

**Table:** 

Title {1}	AGI grade-guided chaiqin chengqi decoction treatment for predicted moderately severe and severe acute pancreatitis (CAP trial): study protocol of a randomised, double-blind, placebo-controlled, parallel-group, pragmatic clinical trial
Trial registration {2a and 2b}.	Chictr.org.cn Registry (ChiCTR2000034325). Registered on 2 July, 2020. https://www.chictr.org.cn/showproj.aspx?proj=55591
Protocol version {3}	The current protocol is version 1, dated December 10, 2019.
Funding {4}	This study is supported by Project of Sichuan Provincial Administration of Traditional Chinese Medicine (Grant No. 2020ZD004) and NZ-China Strategic Research Alliance 2016 Award, Ministry of Science and Technology, China (Grant No. 2016YFE0101800).
Author details {5a}	Zhiyao Chen, West China Centre of Excellence for Pancreatitis, Institute of Integrated Traditional Chinese and Western Medicine, West China Hospital, Sichuan University, Chengdu, China. Xiaonan Yang, West China Centre of Excellence for Pancreatitis, Institute of Integrated Traditional Chinese and Western Medicine, West China Hospital, Sichuan University, Chengdu, China. Jia Guo, West China Centre of Excellence for Pancreatitis, Institute of Integrated Traditional Chinese and Western Medicine, West China Hospital, Sichuan University, Chengdu, China. Tao Jin, West China Centre of Excellence for Pancreatitis, Institute of Integrated Traditional Chinese and Western Medicine, West China Hospital, Sichuan University, Chengdu, China. Ziqi Lin, West China Centre of Excellence for Pancreatitis, Institute of Integrated Traditional Chinese and Western Medicine, West China Hospital, Sichuan University, Chengdu, China. Ping Zhu, West China Centre of Excellence for Pancreatitis, Institute of Integrated Traditional Chinese and Western Medicine, West China Hospital, Sichuan University, Chengdu, China. Jing Li, Chinese Evidence-based Medicine Centre, Cochrane China Centre and MAGIC China Centre, West China Hospital, Sichuan University, Chengdu, China. Ling Li, Chinese Evidence-based Medicine Centre, Cochrane China Centre and MAGIC China Centre, West China Hospital, Sichuan University, Chengdu, China. Xin Sun, Chinese Evidence-based Medicine Centre, Cochrane China Centre and MAGIC China Centre, West China Hospital, Sichuan University, Chengdu, China. Dan Du, West China-Washington Mitochondria and Metabolism Centre, West China Hospital, Sichuan University, Chengdu, China; Institutes for Systems Genetics, Frontiers Science Centre for Disease-related Molecular Network, West China Hospital, Sichuan University, Chengdu, China. Kun Jiang, West China Centre of Excellence for Pancreatitis, Institute of Integrated Traditional Chinese and Western Medicine, West China Hospital, Sichuan University, Chengdu, China. Yanqiu He, West China Centre of Excellence for Pancreatitis, Institute of Integrated Traditional Chinese and Western Medicine, West China Hospital, Sichuan University, Chengdu, China. Fei Cai, West China Centre of Excellence for Pancreatitis, Institute of Integrated Traditional Chinese and Western Medicine, West China Hospital, Sichuan University, Chengdu, China. Lan Li, West China Centre of Excellence for Pancreatitis, Institute of Integrated Traditional Chinese and Western Medicine, West China Hospital, Sichuan University, Chengdu, China. Cheng Hu, West China Centre of Excellence for Pancreatitis, Institute of Integrated Traditional Chinese and Western Medicine, West China Hospital, Sichuan University, Chengdu, China. Qingyuan Tan, West China Centre of Excellence for Pancreatitis, Institute of Integrated Traditional Chinese and Western Medicine, West China Hospital, Sichuan University, Chengdu, China. Wei Huang, West China Centre of Excellence for Pancreatitis, Institute of Integrated Traditional Chinese and Western Medicine, West China Hospital, Sichuan University, Chengdu, China; Liverpool Pancreatitis Research Group, Institute of Systems, Molecular and Integrative Biology, University of Liverpool and Liverpool University Hospitals NHS Foundation Trust, University of Liverpool, Liverpool, United Kingdom. Lihui Deng, West China Centre of Excellence for Pancreatitis, Institute of Integrated Traditional Chinese and Western Medicine, West China Hospital, Sichuan University, Chengdu, China. Qing Xia, West China Centre of Excellence for Pancreatitis, Institute of Integrated Traditional Chinese and Western Medicine, West China Hospital, Sichuan University, Chengdu, China.
Name and contact information for the trial sponsor {5b}	Sichuan Provincial Administration of Traditional Chinese Medicine. No. 15 Yongxing Alley, Chengdu 61000, China.
Role of sponsor {5c}	The study sponsor is not involved in study design, collection, management, analysis or interpretation of the data. The sponsor will not be involved in writing the report of the decision to submit the report for publication.

## Introduction

### Background and rationale {6a}

Acute pancreatitis (AP) is an inflammatory disorder of the pancreas [1]. The common causes of AP such as gallstones, alcohol, hypertriglyceridaemia, endoscopic retrograde cholangiopancreatography (ERCP), and various drugs trigger self-digestion of acinar cells and induce local and systemic inflammation. It has an increasing prevalence with an overall incidence of over 34 affected cases per 100,000 person-years [2]. Most of the patients are uneventful and recover from mild disease (mild acute pancreatitis, MAP), or pull through local complications or transient organ failure defined as moderately severe acute pancreatitis (MSAP). Approximately 15–20% of patients manifesting persistent organ failure (POF) are defined as severe acute pancreatitis (SAP) [3], which are at risk of multiple organ failure (MOF) and the mortality can be up to 50% [4, 5]. Despite the global socioeconomic burden of disease, currently, there are no specific and effective therapeutic agents available to treat AP [6]. According to published guidelines [7, 8], supportive measures, such as analgesia, fluid therapy, nutritional supplementation, and organ support, remain the primary management modalities for AP patients.

Traditional Chinese medicine (TCM), which contains abundant multiple-target biologically active substances, has been broadly used in many countries and regions. Gastrointestinal dysmotility or dysfunction/failure, one of the common and complex complications in AP [9–11], is widely accepted as an important role in the pathogenesis of systemic inflammation and MOF [12–14]. Most TCM herbal formulas, such as dachengqi decoction [15, 16], qingyi decoction [17, 18], and rhubarb [19, 20], are used as an important “gut-centred” therapeutic strategy in the early treatment of AP [21]. Based on TCM theories, we summarised that the key pathogenesis of SAP-MOF is the invasion of “evil heat” to the organs, and its treatment requires “clearing heat, removing toxin, and purging the bowel” in clinical practice [22, 23]. Systematic reviews and meta-analyses of observational studies and trials with small sample sizes have demonstrated the effectiveness of rhubarb-contained TCM in reducing inflammatory markers, organ failure, local complications, and mortality, albeit the included clinical trials are of low quality [24–26].

Chaiqin chengqi decoction (CQCQD) has been modified from dachengqi decoction and used to treat AP in our hospital for over 30 years [27, 28]. The comprehensive approach integrated by TCM and Western medicine improved clinical outcomes and reduced the mortality of SAP to 19.6% [29]. Our previous studies demonstrated that CQCQD can restore intestinal motility or ameliorate systemic inflammation via the toll-like receptor 4 and inflammasome-mediated pro-inflammatory pathways in AP models [23, 30].

Aiming to restoration or maintenance of gastrointestinal function, the Acute Gastrointestinal Injury (AGI) grade-based algorithm will be used to guide the administration of CQCQD [11]. Our trial protocol, informed consent, and other documents were submitted to the Biomedical Research Ethics Committee (BREC) of West China Hospital of Sichuan University in February 2020. After revisions, ethical approval was obtained on 22 June, 2020. The trial was registered with an identifier (ChiCTR2000034325) at chictr.org.cn on 2 July, 2020.

### Objectives {7}

Owing to the lack of sufficient strength of the current evidence, we designed a randomised, double-blind, placebo-controlled, parallel-group trial to provide high-quality evidence of the efficacy of CQCQD for patients with AP (CAP trial).

### Trial design {8}

**Fig. 1:**
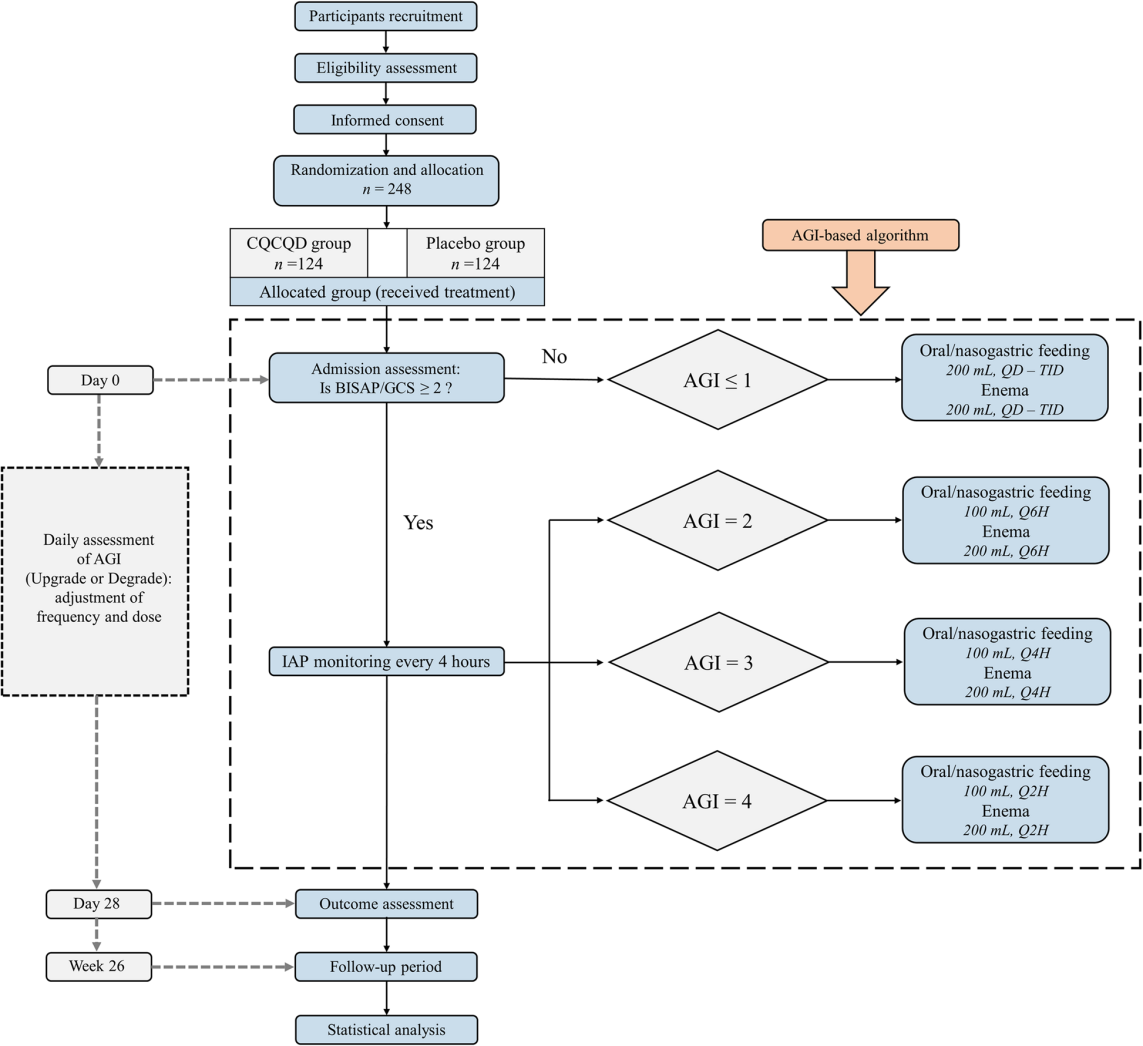
Study flowchart. CQCQD, chaiqin chengqi decoction; AGI, acute gastrointestinal injury; BISAP, Bedside Index of Severity in Acute Pancreatitis; GCS: Glasgow Criteria System; IAP: intra-abdominal pressure; QD: once daily; TID: three times a day; Q6H: every 6 h; Q4H: every 4 h; Q2H: every 2 h

This study is a single-centre double-blind, parallel-design, pragmatic, placebo-controlled randomised trial. The study flowchart is shown in Fig. 1. The trial framework is exploratory.

The protocol follows the recommendations of Standard Protocol Items: Recommendations for Interventional Trials (SPIRIT) guidelines (Additional file 1) [31, 32]. The Pragmatic Explanatory Continuum Indicator Summary Framework-2 (PRECIS-2) (Additional file 2), a validated tool for decision making and discussion of trial design, was used to help us design this pragmatic trial and judge whether these design decisions were fit for our purpose of designing a pragmatic trial [33]. The Consolidated Standards of Reporting Trials (CONSORT) has been used as frameworks of methodology to design this protocol [34].

## Methods: participants, interventions and outcomes

### Study setting {9}

Patients with AP will be recruited through the Pancreatitis Centre Ward of West China Hospital. Eligible patients will be randomly assigned to the treatment or control group with the ratio of 1:1.

### Eligibility criteria {10}

The CAP trial focuses on eligible patients with predicted MSAP and SAP who are admitted within 72 h of onset. A patient will be considered eligible if he/she meets the inclusion criteria and does not meet any of the exclusion criteria.

#### Inclusion criteria

Patients will be included if they fulfil all the following criteria:

1. Meet the diagnostic criteria of AP [3], which fulfil two of the following three items: typical symptoms and signs of abdominal pain, elevated serum amylase and/or lipase at least three times the upper limit of normal, and AP characteristic image findings.
2. Aged between 18 and 75 years, male or female.
3. Admitted to Pancreatitis Centre Ward within 72 h of disease onset.
4. Written informed consent obtained.

#### Exclusion criteria

Patients with any of the following conditions will be excluded:

1. Known pregnant or lactating at admission.
2. The presence of severe organ failure identified using Sequential Organ Failure Assessment (SOFA) scoring by a score of > 3 for individual organs (respiratory, cardiovascular, or renal systems) at admission [35].
3. The predicted MAP defined as a Harmless Acute Pancreatitis Score (HAPS) > 2 at admission [36].
4. Known malignant tumour or having radiotherapy and chemotherapy within 6 months.
5. Known gastrointestinal perforation, bleeding, and mechanical ileus at admission.
6. Acute attack of chronic pancreatitis.
7. Known history of neoplasm in duodenal or biliopancreatic systems.
8. Known history of pancreatic resection.
9. Known undergoing other clinical trials.
10. Known taking TCM orally before enrolment.

### Who will take informed consent? {26a}

Before enrolment, consents for this study are obtained from each patient or his/her next of kin with full information regarding the possible adverse effects of the experimental drug and potential consequences. They will be told about the equal chance of allocation to any one of the two groups before signing the informed consent form. Meanwhile, they will be given enough time to decide whether they join the trial or not. At last, patients will be included voluntarily by signing written informed consent by research assistants.

### Additional consent provisions for collection and use of participant data and biological specimens {26b}

On the consent form, participants will be asked whether they agree to the use of their data and biological specimens. Participants will also be asked to consent to the research team sharing relevant data with regulatory authorities, as appropriate. No identifying images or other personal or clinical details of participants are presented here or will be presented in reports of the trial results. Informed consent forms are available, on request, from the corresponding authors.

## Interventions

### Explanation for the choice of comparators {6b}

#### Conventional treatment regimen

All patients will receive standard treatment according to recently published guidelines [7, 8], including fluid therapy, nutritional supplementation, routine medical treatment such as proton pump inhibitors, analgesia and antibiotics as indicated, organ supportive therapy, and ERCP if necessary. Nasogastric and annal tubes will be used for patients with intra-abdominal hypertension (IAH) or abdominal compartment syndrome (ACS). For patients with persistent ACS or new-onset organ failure, percutaneous drainage of ascites and surgical abdominal decompression will be considered [37, 38]. When pancreatic infection occurs, either a surgical or endoscopic step-up approach considering the location of the necrotic collection and the technical availability will be applied.

#### Study herbal formulation

The prescription of CQCQD was developed by this research team and standardised to contain 13 Chinese medicines, the contents of which can be found in Table 1.

**Table 1:** The ingredients list of standardised Chinese herbal medicine prescription of CQCQD

Ingredients	Latin name	Plant name	Weight (g)
Dahuang	*Rhei Radix et Rhizoma*	*Rheum palmatum* L.	15
Houpo	*Magnoliae Officinalis Cortex*	*Magnolia officinalis* Rehd. et Wils.	15
Zhizi	*Gardeniae Fructus*	*Gardenia jasminoides* Ellis	15
Zhuyechaihu	*Bupleuri Radix*	*Bupleurum chinese* DC.	15
Huangqin	*Scutellariae Radix*	*Scutellaria baicalensis* Georgi	15
Chuanxiong	*Chuanxiong Rhizoma*	*Ligusticum chuanxiong Hort.*	15
Honghua	*Carthami Flos*	*Carthamus tinctorius* L.	10
Zhishi	*Aurantii Fructus Immaturus*	*Citrus aurantium* L.	15
Yanhusuo	*Corydalis Rhizoma*	*Corydalis yanhusuo* W. T. Wang	15
Muxiang	*Auklandiae Radix*	*Aucklandia lappa* Decne.	15
Chishao	*Paeonia Radix Rubra*	*Paeonia veitchii* Lynch	15
Gancao	*Glycyrrhizae Radix et Rhizoma*	*Glycyrrhiza uralensis Fisch.*	3
Mangxiao	*Natrii Sulfas*	Na_2_SO_4_ • 10H_2_O	30

Due to the absence of a positive control drug for AP and the difficulty in preparing a placebo for the decoction, we will utilise a low-dose arm administering 10% CQCQD of similar colour and odour by adding dextrin and colourant, which intend to mimic the placebo group as a control [39]. Generally, the selection of control in a pragmatic trial is usually based on conventional or standard treatment, and placebo is not routinely used as a control. However, the placebo is applied in our trial for following the reasons: (1) The standard treatment for AP including analgesia and fluid therapy is needed in all AP patients and cannot be used only in the control group. (2) Placebo control is designed in our trial to minimise the expectant effect of participants and researchers, as well as to control bias caused by psychological factors from researchers, participants, and evaluators.

The raw herbal drugs with quality certificates will be supplied by Chengdu Kangmei Pharmaceutical Company Limited, Co., Ltd., China. Quality-controlled CQCQD and placebo decoctions will be prepared by the Department of Pharmaceutical Preparation of West China Hospital using the Good Manufacturing Practice standards, according to the 2005 Edition of the Chinese Pharmacopoeia. The high-performance liquid chromatography–mass spectrometry method is used for the quality control of CQCQD. The preparation of the CQCQD and placebo is prepared by two independent pharmacists. During the procedure, a double check will be made by the pharmacist and the researcher. Finally, CQCQD and placebo decoctions will be labelled with computerised numbers to prevent the intervention and control from mixing up. The study decoction preparations will be transported to the inpatient ward and dispensed by two individual research assistants. Researchers will provide the study decoction solution for free. Study investigators and subjects do not know which decoction preparation will be used.

### Intervention description {11a}

#### Administration of randomised drugs guided by the AGI-based algorithm

The AGI grade has been widely used to assess gastrointestinal injury in patients with acute critical illness [11, 40]. Evidence has shown that the AGI grading system is useful for identifying the severity of gastrointestinal dysfunction/failure in AP [41]. In our study, the participants will receive randomised drugs guided by the AGI-based algorithm (Fig. 1).

1. Initiation: If the Bedside Index of Severity in Acute Pancreatitis (BISAP) score or Glasgow Criteria System (GCS) < 2 at allocation [42, 43], the patients will empirically receive CQCQD or placebo 200 mL oral intake (or intragastric infusion) and enema, 3 times a day. If the patients have a BISAP/GCS score ≥ 2 at allocation, they will be required to receive urethral catheterisation to monitor intra-abdominal pressure (IAP) every 4 h, which is the key index in AGI grade. The dosage and frequency of decoction is determined by AGI grading according to the following criteria: (1) if AGI grade = 1, 200 mL CQCQD or placebo is administered orally (or intragastrical infusion) and enema, 3 times a day; (2) if AGI grade = 2, CQCQD or placebo is given 100 mL orally (or intragastrical infusion) and 200 mL enema, every 6 h; (3) if AGI grade = 3, CQCQD or placebo are given 100 mL orally (or intragastric infusion) and 200 mL enema, every 4 h; (4) if AGI grade = 4, CQCQD or placebo is given 100 mL orally (or intragastric infusion) and 200 mL enema, every 2 h. If the patient with a BISAP/GCS score ≥ 2 at allocation rejects urethral catheterisation, the initial administration of the randomised drug is in accordance with AGI grade 1, and the conditions will be reassessed for further judgement.
2. Continuation: AGI grade is evaluated daily at 7–8 am by the researchers. The dosage and frequency of CQCQD or placebo are determined by the corresponding AGI grade at the time points.
3. Termination: CQCQD or placebo is administered for at least 5 days and no more than 14 days. IAP monitoring will be terminated if it is less than 15 mmHg for two consecutive days. CQCQD or placebo will be terminated, if AGI grade < 1 for two consecutive days, or normally oral refeeding without discomfort. Other conditions will be determined by clinical treatment leaders and principal investigators.
4. The upgrade principle for patients with AGI grade 1 initially or who reject urethral catheterisation: (1) the aggravation of abdominal distension and constipation; (2) the occurrence of new organ dysfunction; (3) the deterioration of the original organ function; and (4) other conditions will be assessed and determined by clinical treatment leaders and principal investigators.

### Criteria for discontinuing or modifying allocated interventions {11b}

The patients are withdrawal if they meet any of the following criteria:

1. Having manifestations of gastrointestinal perforation, bleeding, mechanical ileus, severe haemorrhoids, and anal fissure which require restricted fast and enema.
2. Quitting voluntarily.
3. Adverse medical events occurring during the study, such as severe hypernatremia, intolerance to critically worsening conditions, or other reasons as judged by the investigators, which might make the patient unlikely to complete the study.

### Strategies to improve adherence to interventions {11c}

Although flex of experimental intervention adherence is used (Additional file 2), efforts will be made to improve adherence to intervention: patient education (educate patients about the necessity of urethral catheterisation to monitor IAP, educate their individual risk of disease-related complications) and medication-taking reminders (researchers monitor and reminder the frequencies and volume of medication).

### Relevant concomitant care permitted or prohibited during the trial {11d}

During hospitalisation, the patients are not allowed to use other Chinese medicines and alternative therapies (e.g. herbal extract injection, acupuncture, abdominal ultrasound therapy). Drugs for inhibitory gastrointestinal motility (e.g. catecholamines, sedatives) and prokinetics (e.g. neostigmine, domperidone, metoclopramide, erythromycin) are not used routinely unless the patients present with AGI grade 4 or other critical conditions suggested by clinical treatment leaders and principal investigators to ensure the safety of the patients. All the additional drugs or therapies must be recorded in case report form (CRF) in detail.

### Provisions for post-trial care {30}

Participants who are discharged and re-admitted within 4 weeks will be regarded as a re-admission for the same episode of care and will be in the same arm as their original allocation. Data of re-admission elsewhere will be collected at follow-up typically at 4 weeks.

### Outcomes {12}

#### Primary outcome measurements

Referring to the existing study results and expert recommendations [6, 28, 29, 44], the duration of respiratory failure (SOFA score ≥ 2 points for respiratory) within 28 days after admission will be served as the primary outcome in the CAP trial.

#### Secondary outcome measurements

I. Secondary outcomes during the index admission

1. The occurrence of new-onset of any organ failure (SOFA score for respiratory, cardiovascular, or renal system ≥ 2 points) or new-onset of POF (organ failure lasts > 48 h). New onset is defined as events that occur after randomisation and not present 24 h before randomisation.
2. Cumulative levels of C-reactive protein (CRP) on days 1, 2, 3, 5, 7, 14, 21, and 28 after admission.
3. Levels of albumin, procalcitonin, interleukin-6 (IL-6), and neutrophil count on day 0, day 3, day 5, day 7, and day 14.
4. Daily visual analogue scale (VAS) score of pain [45].
5. Daily Pancreatitis Activity Scoring System (PASS) score within the 1st week after enrolment [46].
6. Duration (days) of Systemic Inflammatory Response Syndrome (SIRS ≥ 2 points) [47].
7. Duration (days) of fasting.
8. The requirement of the anal tube.
9. The requirement of prokinetic agents.
10. Duration (days) of gastrointestinal decompression.
11. Duration (days) of IAH and ACS.
12. The receipt of respiratory support and renal replacement therapy.
13. Length of intensive care unit (ICU) stays.
14. Local pancreatic injury by contrast-enhanced computerised tomography (CECT) on days 7 and 14.
15. Potential safety signals.
16. Length of hospital stay.
17. In-hospital cost.

II. Secondary outcomes within 26 weeks after enrolment

1. The requirement for catheter drainage or necrosectomy.
2. Infected pancreatic necrosis.
3. All-cause mortality.
4. Self-report health situation by five-level EuroQol five-dimensional (EQ-5D-5L) [48].

### Participant timeline {13}

The participant timeline is shown in Table 2.

**Table 2:**
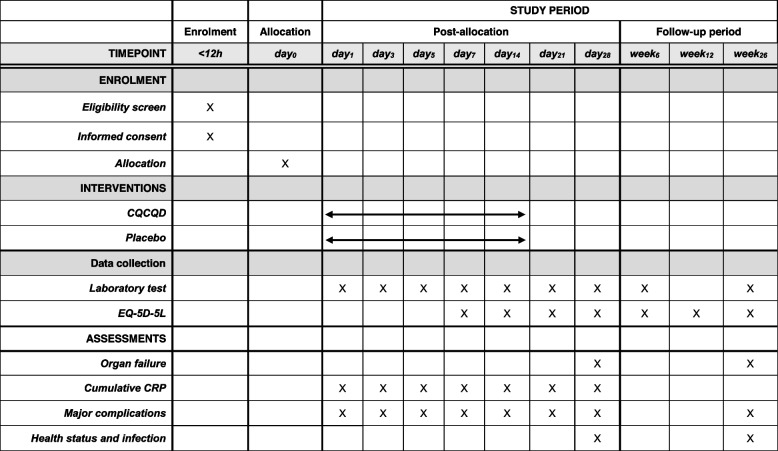
Study schedule of the CAP trial

*CQCQD* chaiqin chengqi decoction, *EQ-5D-5L* five-level EuroQol five-dimensional questionnaire, *CRP* C-reactive protein

### Sample size {14}

The sample size calculation was based on the following assumptions: a reduction in duration of respiratory failure of 2 days in the treatment group, and a standard deviation of 4.5 days for each group [17, 29]. Assuming that the significance level is 0.05 and the study power is 0.8 (two-sided *α* = 0.05, *β* = 0.2), we projected a sample size of 111 patients in each group using Power Analysis & Sample Size software (PASS statistic software, version 11.0.7, NCSS). Considering a 10% dropout rate, a total of 248 patients will be recruited in this trial with 124 patients for each group.

### Recruitment {15}

Patients admitted to the Emergency Department of West China Hospital will be primarily screened via the symptoms of abdominal pain and onset time by research nurses. Patients with a diagnosis of AP will be admitted to Pancreatitis Centre Ward for further recruitment.

## Assignment of interventions: allocation

### Sequence generation {16a}

After the completion of screening measurements and the acquisition of signed consent, eligible participants will be randomised in a 1:1 ratio to either the treatment or control group. The randomisation code will be automatically created by computer using Analytics Software & Solutions software (SAS, version 9.3, SAS Institute Inc, USA).

### Concealment mechanism {16b}

Patients will be randomised using sequentially numbered, opaque sealed envelopes, which is the most accessible and straightforward method of maintaining allocation concealment. A group of independent assessors will interview the patients and perform the screening.

### Implementation {16c}

Statistician (Ms. Ping Zhu) generated the allocation sequence. Research assistants will continue the enrolment process by screening eligible patients according to the inclusion and exclusion criteria and assigning participants to interventions.

## Assignment of interventions: blinding

### Who will be blinded {17a}

All the patients, researchers, and statisticians in this clinical study will be blinded to the treatment assignment and unaware of the actual medications.

### Procedure for unblinding if needed {17b}

The study blinding will only be broken in a medical emergency when the treating physician believes that the administration of the study drug is associated with the emergency.

## Data collect and management

### Plans for assessment and collection of outcomes {18a}

According to the study schedule shown in Table 2, data will be collected during the index admission and 26 weeks after enrolment. If a participant wishes to discontinue the study drug or the treating physician believes a participant should discontinue the study drug due to medical conditions, the investigator will communicate with the participant and the treating physician to obtain the reasons and record in CRF. Further follow-up will still be performed unless the participant withdraws from the trial.

### Plans to promote participant retention and complete follow-up {18b}

Participants are encouraged to visit the researchers in outpatients on weeks 6, 12, and 26 after enrolment, but they will decide a relatively flexible visiting time or follow-up via telephone. During the follow-up period, participants will receive personalised telephone and online counselling with doctors so that they will receive qualified medical care by the research group. They can emergently contact the researcher (Dr. Lihui Deng) if they have discomfort. Incentives to promote adherence will also be provided (e.g. free registration fees, during follow-up transportation allowances, admission priority due to recurrence or complications of pancreatitis).

### Data management {19}

The researchers involved will receive restricted training to ensure that the study process is detailed. Data collection will use a CRF and include source-verifiable data from patient records. CRF will be anonymised and contain no individual patient-identifiable information. Paper copies of the CRF will be stored in a locked cabinet in the Chief Investigator’s office in West China Hospital. All data will be input by the primary investigator or nominated investigators approved by the primary investigator, and a double check will be performed by the research coordinator.

### Confidentiality {27}

At the time of recruitment, an unrelated sequence of characters will be used to replace the participant’s identifiable information. The data will be stored in the Pancreatitis Centre Information Records Office and will only be accessible to researchers, data monitors, and the BREC of West China Hospital.

### Plans for collection, laboratory evaluation, and storage of biological specimens for genetic or molecular analysis in this trial/future use {33}

Blood samples of participants will be collected on admission and 1, 3, 5, and 7 days after admission, and the samples will be transferred and handled by professional biobank technicians. Finally, serum, plasma, and white blood cell samples will be stored in West China Biobanks, certified by the China Human Genetic Resources Management Office (No. [2016]406 and No. [2022]BC0040).

## Statistical methods

### Statistical methods for primary and secondary outcomes {20a}

Data cleaning and statistical analysis will be performed by a statistician blinded to the whole trial process using the latest version of SAS software. The results of the data will be expressed as the means with ranges, medians with interquartile ranges, or numbers of patients with percentages. The comparison between the individualised CQCQD and control group is the primary interest in this study. Continuous variables are compared using the independent-sample *T* test or the Mann–Whitney *U* test (for non-normal distributions). Categorical variables are compared with the chi-squared test or Fisher’s exact test. Statistical tests will be two-sided, and *P* values < 0.05 will be considered significant.

### Interim analyses {21b}

Interim analysis is not planned in the CAP trial.

### Methods for additional analyses (e.g. subgroup analyses) {20b}

The detailed analysis strategies for subgroup analyses by the severity of AP (severe and nonsevere), age (dichotomised at 60 years old), and aetiologies of AP (hypertriglyceridaemic and non-hypertriglyceridaemic) will be compared using appropriate summary statistics (according to its distribution).

### Methods in analysis to handle protocol non-adherence and any statistical methods to handle missing data {20c}

The data will be analysed based on the full analysis set (FAS), per-protocol set, and safety set. FAS analysis is the basic principle of intention-to-treat, including all the enrolled patients in the trial. Analysis will follow intention-to-treat principles with patients analysed according to randomisation and irrespective of actual use or compliance with the algorithm. The per-protocol analysis is the subset of FAS analysis, which just includes patients who finish the trial completely. The safety set analysis is the set of patients who receive the treatment and have safety assessment. Missing values will be addressed by multiple imputation, having appropriately explored the missingness mechanism and in accordance with good practice.

### Plans to give access to the full protocol, participant-level data, and statistical code {31c}

Final data of the trial that support the findings of this study are available from the corresponding author upon reasonable request.

## Oversight and monitoring

### Composition of the coordinating Centre and trial steering committee {5d}

The trial steering committee consists of four members, who will hold meetings monthly and audit the trial. Dr. Qing Xia, as the primary investigator, will be responsible for the overall management of the trial. Dr. Lihui Deng has expertise in clinical trials and will be the clinical lead in the trial. Dr. Wei Huang has expertise in biological specimen management and will lead the biobank platform for the trial. Dr. Dan Du is an expert who will ensure the quality control of CQCQD.

### Composition of the data monitoring committee, its role and reporting structure {21a}

An Independent Data and Safety Monitoring Committee (IDSMC) consists of two clinical experts and one statistician, who are responsible for overseeing the safety and quality of the data. The committee is independent and has no competing interests with the research. The IDSMC will consider protocol adherence, trial withdrawal, and safety monitoring and will make recommendations for continuation of the trial.

### Adverse event reporting and harms {22}

Adverse events (AEs) are defined in accordance with the National Cancer Institute-Common Terminology Criteria for Adverse Events. The common aberrations in symptoms, signs, and laboratory values due to the severity of the underlying disease and the impact of standard therapies will not necessarily constitute an AE unless they require significant intervention or are of concern in the investigator’s clinical judgement. If severe adverse events (SAEs) happen that threaten the patients’ safety, the researcher should stop the study at once, cancel the blinding, and take any measures to rescue the patients’ lives who experience SAE.

All details of any AE/SAE will be documented and reported during the treatment and the follow-ups. Furthermore, SAE will be reported to the principal investigator and the BREC immediately to decide whether the patient should withdraw from the trial.

### Frequency and plans for auditing trial conduct {23}

The BREC of West China Hospital will audit the trial process every 3 months.

### Plans for communicating important protocol amendments to relevant parties (e.g. trial participants, ethical committees) {25}

Any amendments to the protocol will be submitted to the BREC, who will review and approve the amendments. The protocol amendments will be updated in the clinical trial registry accordingly. In addition, participants will be informed of the protocol amendments.

### Dissemination plans {31a}

The results of the trial will be disseminated in peer-reviewed journal publications and conference presentations.

## Discussion

Over the past 30 years, the Chinese herbal formula CQCQD has been widely used as an adjunctive therapy of conventional comprehensive treatment for AP in our hospital. A plethora of basic research studies from our group have revealed that CQCQD reduced pro-inflammatory indices and improved pancreatic and multiple organ injuries in experimental AP models [23, 49–53]. However, high-quality evidence from clinical studies to demonstrate its efficacy in AP patients is still lacking. Therefore, the CAP trial is designed to investigate the efficacy of CQCQD in MSAP/SAP, who are most voluntary to gastrointestinal and respiratory failures with substantial morbidities and mortality of AP.

The CAP trial is conducted at Pancreatitis Centre of West China Hospital, an “all-in-one” pancreatitis centre with 74 routine open beds designated for AP patients who are managed by a TCM-led, gastroenterologists and pancreatic surgeons incorporated multiple-discipline team. It is one of the largest single tertiary referral centres in the world admitting over 1800 cases of AP annually. This specific regimen of CQCQD treatment for AP, which limited the extension and application of this pattern to international centres and some other domestic centres. Therefore, the CAP trial is designed as a single-centre study to assure the homogeneity of the study.

Quality control for CQCQD plays a vital role in the trial. Hundreds of characteristic phytochemicals contained in compound formulas pose a challenge for developing robust quality assessment metrics. In a previous study [50], we successfully constructed a multi-strategy-based analytical method and identified potential quality-markers based on drug properties and effect characteristics. The preparation of the decoction is performed by our Department of Pharmaceutical Preparation according to standard operating procedures. Despite qualified materials used for CQCQD preparation, the quality of CQCQD will be assessed by the appropriate content range of the markers. This feasible platform lays the solid foundation for the CAP trial.

Outcome determination is of great importance for the trial. A recent evidence-based study suggested that trials in participants with AP should consider complications or health-related quality of life, but not mortality, as primary outcomes [6]. In the CAP trial, the duration of respiratory failure will be served as the primary endpoint. Respiratory failure constitutes a high proportion of organ failure in AP [4, 29]. In the Zangfu theory of TCM, the lung and the large intestine are interior-exteriorly related. Based on this theory, the method “purging the bowel” could potentially treat pulmonary disease [54]. The CAP trial will employ the AGI-guided algorithm for CQCQD administration to shorten the duration of respiratory failure in the early stage as a primary endpoint for SAP. Most of the secondary endpoints are registered within the index admission and follow-up patients for 6 months.

In conclusion, the CAP trial is the first pragmatic randomised controlled trial evaluating the efficacy of CQCQD in the early management of gastrointestinal and respiratory failures in predicted MSAP/SAP patients. If the CAP trial will be successfully implemented, the results would provide high-quality evidence to appraise the effects of CQCQD in AP patients.

## Trial status

The current protocol is version 1, dated December 10, 2019. The trial started to recruit patients on August 1, 2020, and is anticipated to be completed on June, 2023.

## Supplementary Information

**Additional file 1.** SPIRIT 2013 Checklist: Recommended items to address in a clinical trial protocol and related documents.

**Additional file 2.** PRECIS-2 scores of chaiqin chengqi decoction for predicted moderately severe and severe acute pancreatitis (CAP trial) domains.

## Data Availability

Data will be stored in a secure database that can only be accessed by the study chief investigator.

## Abbreviations

AP: Acute pancreatitis
ERCP: Endoscopic retrograde cholangiopancreatography
MAP: Mild acute pancreatitis
MSAP: Moderately severe acute pancreatitis
POF: Persistent organ failure
SAP: Severe acute pancreatitis
TCM: Traditional Chinese medicine
MOF: Multiple organ failure
CQCQD: Chaiqin chengqi decoction
CAP: CQCQD for AP
AGI: Acute Gastrointestinal Injury
BREC: Biomedical Research Ethics Committee
PRECIS-2: Pragmatic Explanatory Continuum Indicator Summary Framework-2
SPIRIT: Standard Protocol Items: Recommendations for Interventional Trials
CONSORT: Consolidated Standards of Reporting Trials
SOFA: Sequential Organ Failure Assessment
MAP: Mild acute pancreatitis
HAPS: Harmless Acute Pancreatitis Score
BISAP: Bedside Index of Severity in Acute Pancreatitis
GCS: Glasgow Criteria System
IAP: Intra-abdominal pressure
IAH: Intra-abdominal hypertension
ACS: Abdominal compartment syndrome
CRF: Case report form
VAS: Visual analogue scale
CECT: Contrast-enhanced computerised tomography
CRP: C-reactive protein
ICU: Intensive care unit
SIRS: Systemic Inflammatory Response Syndrome
PASS: Pancreatitis Activity Scoring System
IL-6: Interleukin-6
EQ-5D-5L: Five-level EuroQol five-dimensional
IDSMC: Independent Data and Safety Monitoring Committee
AEs: Adverse events
SAEs: Severe adverse events
FAS: Full analysis set
