# Supplementary material for: AGI grade-guided chaiqin chengqi decoction treatment for predicted moderately severe and severe acute pancreatitis (CAP trial): study protocol of a randomised, double-blind, placebo-controlled, parallel-group, pragmatic clinical trial
Source: Trials. 2022 Nov 8;23:933. doi: 10.1186/s13063-022-06792-x (PMC9644559; doi:10.1186/s13063-022-06792-x)
Supplement: Supplementary file 2 — Additional file 2. PRECIS-2 scores of chaiqin chengqi decoction for predicted moderately severe and severe acute pancreatitis (CAP trial) domains. [file 13063_2022_6792_MOESM2_ESM.pdf]

Additional file 2

Table 1. PRECIS-2 scores of chaiqin chengqi decoction for predicted moderately severe and severe acute pancreatitis (CAP trial) domains

	Domain	Score	Rationale
1	Eligibility Criteria	4	Patients diagnosed with acute pancreatitis will be initially recruited. The time interval of the onset is limited within 72 hours from abdominal pain in order to focus on the early treatment of CQCQD. The primary endpoint of the trial is the duration of respiratory failure, but mild patients do NOT develop organ failure. For this reason, we intend to exclude mild acute pancreatitis and include the predicted moderate and severe patients. To exclude the patients less than 18 or over 70 years old, severe organ failure upon admission, pregnancy and so on, we aim to ensure the safety of the participants with this critically illness. Other criteria will not restrict the participants who conform to study aim.
2	Recruitment Path	5	As acute pancreatitis is an acute illness, we start to consecutively screen and recruit AP patients in Emergency Department. There is no other recruitment path needing extra efforts.
3	Setting	3	The trial is sponsored in a single tertiary referral center, which is based on multiple-discipline team (MDT) system combined by traditional Chinese and Western medicine the and endeavor to widespread CQCQD use in China.
4	Organization intervention	5	The trial employs the same groups of clinicians and nurses as usual clinical service. It does need additional training and other certification for the staff.
5	Flex of experimental intervention – Delivery	5	Full flexibility. Although the administration of CQCQD is guided by Acute Gastrointestinal Injury (AGI) grade-based algorithm, the treatment of critical patients will also follow the advices from clinical treatment leaders and principal investigators.
6	Flex of experimental intervention – Adherence	4	Full flexibility. Patients are required to orally intake the daily dose of CQCQD and can decide the frequency and single dose according to their own conditions (such as nausea, vomit,

			bloating). They also have right to decide the urethral catheterization, and we propose the corresponding protocol for those who refuse the urethral catheterization and intra-abdominal pressure measurement.
7	Follow up	4	Patients are encouraged to visit the researchers in outpatients on week 6, 12 and 26 after enrolment, but they will decide a relatively flexible visiting time or follow up via telephone.
8	Outcome	5	The outcome measures are closely relevant to participants. The primary endpoint is duration of respiratory failure, which poses highest incidence among organ failure and is of importance for severity determinant and prognosis.
9	Analysis	3	Intention-to-treat (ITT) and per-protocol (PP) analysis will be used in the trial.

Figure 1. PRECIS-2 wheel for chaiqin chengqi decoction for predicted moderately severe and severe acute pancreatitis (CAP trial).

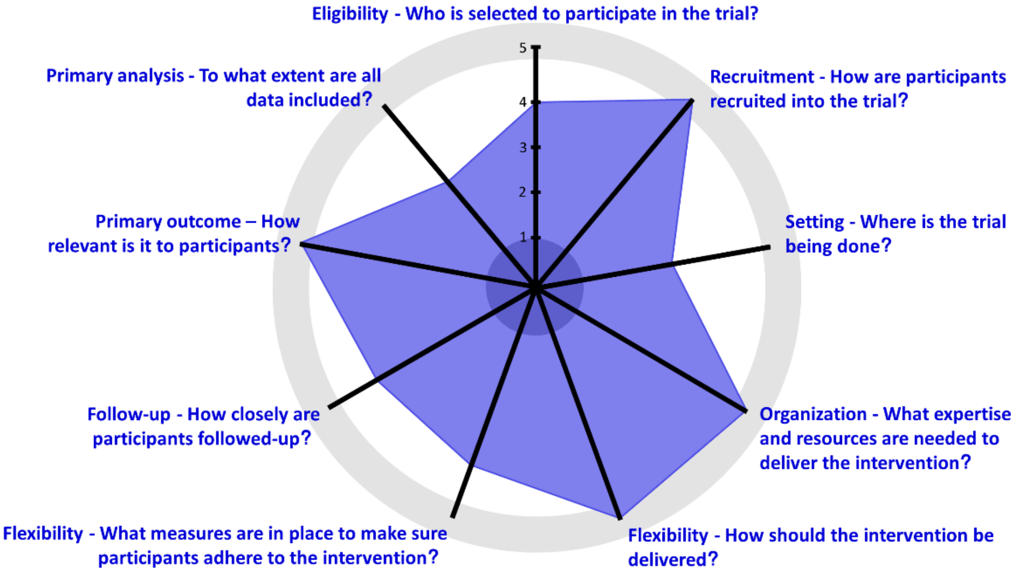

Scores of the nine PRECIS-2 domains are: Eligibility: 4, Recruitment: 5, Setting: 3, Organization: 5, Flexibility (delivery): 5, Flexibility (adherence): 4, Follow-up: 4, Primary outcome: 5, Primary analysis: 3.
